# Corticosterone stimulates synthesis of 2-arachidonoylglycerol via putative membrane-bound glucocorticoid receptors and inhibits GABA release via CB1 cannabinoid receptors in the ventrolateral periaqueductal gray

**DOI:** 10.1016/j.molpha.2025.100058

**Published:** 2025-06-21

**Authors:** Basile Coutens, Courtney A. Bouchet, Lorenzo C. Patti, Kylie B. McPherson, Bethany S. Boston, David C. Jewett, Susan L. Ingram

**Affiliations:** 1 Department of Anesthesiology, University of Colorado, Anschutz Medical Campus, Aurora, Colorado; 2 Department of Biomedical Sciences, Colorado State University, Fort Collins, Colorado; 3 Department of Psychology, University of Wisconsin-Eau Claire, Eau Claire, Wisconsin

**Keywords:** Corticosterone, Diacylglycerol lipase, Endocannabinoid, Periaqueductal gray, Protein kinase A

## Abstract

The ventrolateral periaqueductal gray (vlPAG) plays a critical role in pain modulation. GABAergic neurotransmission within the vlPAG regulates the descending pain pathway. This study investigates the mechanisms through which corticosterone (CORT) modulates GABA release in the vlPAG via putative membrane-associated glucocorticoid receptors (mbGRs). Superfusion of CORT decreases evoked inhibitory postsynaptic currents in a mbGR- and CB1 cannabinoid receptor (CB1R)-dependent manner. Using a depolarization-induced suppression of inhibition protocol to test the effects of CORT on the endocannabinoid system, we find that CORT-mediated signaling enhances 2-arachidonoylglycerol synthesis that is inhibited by the diacylglycerol lipase inhibitor, DO34. CORT prolongs CB1R activation through a G*α*_s_ and protein kinase A-dependent pathway, whereas early depolarization-induced suppression of inhibition-initiated endocannabinoid activation of CB1Rs is independent of protein kinase A. These results highlight the critical role of CORT in the vlPAG in engaging endocannabinoid pathways to inhibit GABA release. The results indicate that CORT activation of putative mbGRs promote activation of the descending pain modulatory pathway through CB1R-mediated inhibition of GABA release in the vlPAG.

**Significance Statement:**

This study provides evidence that corticosterone activates putative membrane glucocorticoid receptors to increase levels of 2-arachidonoylglycerol to activate presynaptic CB1 cannabinoid receptors. These findings reveal mechanisms by which stress modulates the ventrolateral periaqueductal gray and the descending pain circuit.

## Graphical abstract

**Figure:**
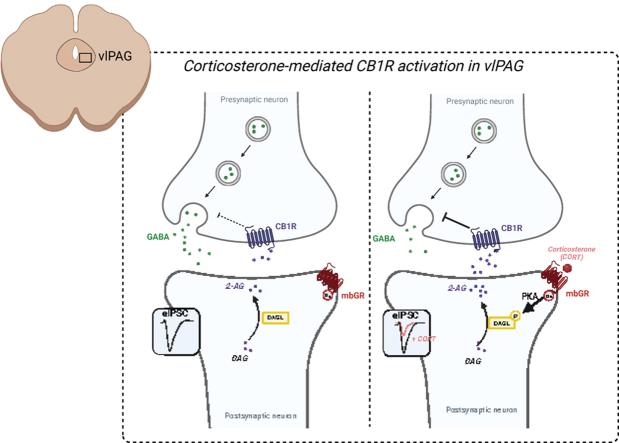

## 1 Introduction

The hypothalamic-pituitary-adrenal (HPA) axis is the major neuroendocrine system that regulates stress responses via glucocorticoid hormones.^1^ Circulating cortisol, or corticosterone (CORT) in rodents, influences different physiological and behavioral functions by acting on the central nervous system, particularly in brain regions that express glucocorticoid receptors (GRs) and are associated with stress, anxiety, and pain.^2^^,^^3^ Under stress conditions, CORT triggers rapid increases in the endocannabinoid 2-arachidonoylglycerol (2-AG)^4^, ^5^, ^6^, ^7^ via putative membrane-bound glucocorticoid receptors (mbGRs).^8^^,^^9^ In contrast to classical nuclear GRs, mbGRs mediate rapid, nongenomic responses.^10^^,^^11^ In the hypothalamus, CORT increases 2-AG levels that inhibit glutamate release and indirectly facilitate GABA release,^12^ whereas other studies in the basolateral amygdala provide evidence that 2-AG directly inhibits GABA transmission.^13^^,^^14^ Thus, the effects of CORT-mediated endocannabinoid release are specific to different brain regions.

The ventrolateral periaqueductal gray (vlPAG) is an important brain area involved in the descending pain modulatory pathway.^15^^,^^16^ Both exogenous cannabinoids and endocannabinoids in the PAG modulate pain and stress.^17^, ^18^, ^19^, ^20^ In addition, both pain^21^, ^22^, ^23^, ^24^ and stress^25^, ^26^, ^27^ modulate levels of endocannabinoids in the PAG. The endocannabinoid system includes 2 major endocannabinoids, 2-AG and anandamide, which are released by the postsynaptic neuron and bind presynaptic cannabinoid receptors leading to a decrease of neurotransmitter release.^28^^,^^29^ Inhibition of enzymes that break down endocannabinoids reveals that both endocannabinoids can be generated and released in the PAG.^30^ Endocannabinoids and exogenous CB1 cannabinoid receptor (CB1R) agonists inhibit GABA and glutamate synaptic neurotransmission in the vlPAG.^22^^,^^30^, ^31^, ^32^, ^33^, ^34^, ^35^ Interestingly, inhibition of glutamatergic excitatory postsynaptic currents (EPSCs) is more evident in vlPAG slices cut in the horizontal plane^31^ compared with the coronal plane^35^^,^^36^ which may explain differences in results between studies regarding the balance between cannabinoid actions on inhibitory versus excitatory neurotransmission.

GRs are expressed in the vlPAG^37^; however, the role of CORT in regulating endocannabinoids and synaptic inhibition within the vlPAG remains unexplored. The present study investigates the mechanisms underlying CORT modulation of GABA neurotransmission in the vlPAG. The results indicate that CORT increases 2-AG synthesis via G*α*_s_-coupled GRs and protein kinase A (PKA) signaling. Through this pathway, CORT could modulate nociceptive thresholds and the descending pain pathway, highlighting an intricate relationship between the HPA axis and the endocannabinoid system within the vlPAG.

## 2 Materials and methods

### 2.1 Animals

Adult male and female Sprague-Dawley rats (3–11 weeks old) were used for all experiments. All procedures were performed in strict accordance with the NIH (National Research Council) Guide for the Care and Use of Laboratory Animals as adopted by the Institutional Animal Care and Use Committee of the University of Colorado Anschutz Medical Campus and comply with ARRIVE guidelines.

### 2.2 Drugs

SR141716A (rimonabant; RIM; Cayman Chemical), WIN 55,212-2 (WIN; Cayman Chemical), corticosterone (CORT; Tocris), 11b-(4-dimethyl-amino)-phenyl-17bhydroxyl-17-(1-propynyl)-estra-4,9-dien-3-one (RU486; Tocris), and DO34 (2-AG synthesis inhibitor, MedChemExpress) were dissolved in DMSO, aliquoted, and stored at –20 °C. DMSO was dissolved 1:10,000 DMSO:artificial cerebrospinal fluid (aCSF) in all cases and added to the appropriate baseline (vehicle) measurements. NBQX and gabazine (Tocris) were dissolved in milliQ water and stored at 4 °C. Dexamethasone conjugated to bovine serum albumin (DEX-BSA; Steraloid) is a GR agonist that is membrane impermeable. This covalent conjugation is considered irreversible and is widely used to restrict glucocorticoid action to membrane-associated receptors, minimizing genomic effects.^38^^,^^39^ The DEX-BSA has a steroid-to-BSA molar ratio of 37:1. To determine the mechanism of action of CORT on 2-AG synthesis, a PKA inhibitor (PKI, Tocris) was used directly in the internal solution in recording electrodes or via bath application. Finally, a peptide inhibitor [Gs-myr; Seq:Myr-MGQRMHLRQYELL, GenScript^40^] was used to block G*α*_s_ proteins.

### 2.3 Ex vivo slice preparation

Slices containing the vlPAG were prepared as previously described.^15^^,^^35^ Rats were deeply anesthetized with isoflurane (McKesson), and the brain was rapidly removed and placed in ice-cold aCSF cutting buffer containing the following (in mM): 126 NaCl, 21.4 NaHCO_3_, 22 dextrose, 2.5 KCl, 2.4 CaCl_2_, 1.2 MgSO_4_, and 1.2 NaH_2_PO_4_ (300 mOsm). Slices containing the vlPAG were cut to a thickness of 220 *μ*m on a vibratome (Leica Microsystems) and were transferred to a holding chamber maintained at 32 °C. Slices were oxygenated with 95% O_2_/5% CO_2_ until transfer to the recording chamber on an upright microscope (model BX51WI, Olympus–Evident Scientific) and superfused with oxygenated aCSF maintained at 32 °C.

### 2.4 Whole-cell patch-clamp recordings

Voltage-clamp recordings (holding potential, –70 mV) were made in whole-cell configuration using an amplifier (MultiClamp700B, Molecular Devices), sampled at 2 kHz, and digitized at 5 kHz with the Axon Digidata 1550B (Molecular Devices) using Clampex 11.0.3 software (Molecular Devices). Patch-clamp electrodes were pulled from borosilicate glass (diameter, 1.5 mm; World Precision Instruments) on a two-stage puller (Narishige). Pipettes had a resistance between 2.5 and 4 M*Ω* and were filled with an intracellular pipette solution containing the following (in mM): 140 CsCl, 10 HEPES, 4 MgATP, 3 NaGTP, 1 EGTA, 1 MgCl_2_, and 0.3 CaCl_2_ (pH 7.3, 290–300 mOsm). QX314 (100 *μ*M) was added to the internal solution for evoked inhibitory postsynaptic current (eIPSC) experiments to reduce action potentials in the recording cell. Access resistance was continuously monitored. Recordings in which access resistance changed by 20% during the experiment were excluded from data analysis. A bipolar stimulating electrode (FHC), placed into the vlPAG approximately 200 *μ*m from the recording electrode, was used to deliver 2-millisecond pulses of 100 *μ*A to 10 mA to eIPSCs. A junction potential of 5 mV was corrected during recording. GABAergic eIPSCs were isolated in the presence of glutamate receptor antagonist [2,3-Dioxo-6-nitro-1,2,3,4-tetrahydrobenzo[*f*]quinoxaline-7-sulfonamide disodium salt (NBQX); 5 *μ*M], whereas evoked EPSCs (eEPSCs) were isolated in the presence of gabazine (10 *μ*M). Spontaneous IPSCs (sIPSCs) were recorded in the presence of NBQX (5 *μ*M). Measurements were taken during the final 30 seconds of superfusion of drugs. In experiments using exogenous cannabinoid agonists, or GR agonists/antagonists, only 1 neuron was recorded per slice. Agonists were added to the bath after a stable baseline was achieved in NBQX for at least 5 minutes. Cannabinoid and glucocorticoid agonists were bath-applied for 5–10 minutes, followed by the addition of either a GR antagonist (RU8486) or the CB1R antagonist, RIM, to assess the reversibility and specificity of the observed effects. Multiple eIPSCs were averaged when maximal drug effect was achieved. After each experiment, the lines were washed with 70% ethanol and then rinsed with milliQ water. Percentage of eIPSC inhibition was calculated as 100– [(Avg CORT amplitudes)/(Avg control amplitudes)∗100].

### 2.5 Depolarization-induced suppression of inhibition

After obtaining stable eIPSCs, a protocol for depolarization-induced suppression of inhibition (DSI) collected 2 eIPSCs for baseline measurement, followed by a brief depolarizing step (5 seconds at +20 mV)^35^^,^^41^ before returning to the holding potential (–70 mV). eIPSCs were evoked at 0.2 Hz for 60 seconds following the depolarizing step, and amplitudes were normalized to the average baseline eIPSC amplitudes [Normalized DSI values = [(Avg DSI eIPSC amplitudes/(Avg baseline eIPSC amplitudes)]. Not all cells were sensitive to the DSI protocol, which is consistent with observations from previous experiments and other laboratories.^35^^,^^42^ Therefore, we included cells that did not display inhibition of GABAergic eIPSCs with the DSI protocol.

### 2.6 Statistical analyses

In all electrophysiological experiments, each dataset included recordings from at least 3 rats. For DSI experiments, 2 separate time windows were analyzed: the average of the first 5 eIPSCs (5–25 seconds) following depolarization, and the average of the subsequent 5 eIPSCs (30–50 seconds). Values are presented as the mean ± SEM, and all data points are shown in bar graphs to illustrate variability. Statistical comparisons were made using *t*-test, or one- and two-way ANOVA, as appropriate. In all summary bar graphs, each dot represents an individual cell while the numbers in the bars represent the animal number. When post hoc analysis was appropriate, multiple-comparisons tests were performed and specified in the figure legends. Significance was defined as *P* < .05. All analyses were conducted in GraphPad prism (GraphPad).

The PAG is heterogeneous and contains many cell types that are not discernable in our rat slices^43^ and bipolar stimulating electrodes stimulate neurons and axons of passage indiscriminately when placed into the vlPAG. Thus, each whole-cell patch-clamp experiment is inherently independent and variability arises from activation of synaptic inputs in the area surrounding the bipolar electrodes rather than interanimal differences. Nevertheless, we limit the number of recordings per animal—typically no more than 2 recordings per rat. However, multiple DSI recordings per slice were tested to reduce animal numbers due to the variable nature of the experiments as noted in several other studies.^35^^,^^41^ No differences in significance were noted when using ANOVA and nested ANOVA statistical analyses supporting the interpretation that cells are independent variables in these studies.

## 3 Results

### 3.1 CORT-activated GRs indirectly inhibit GABA release via CB1R activation in vlPAG

**Fig. 1:**
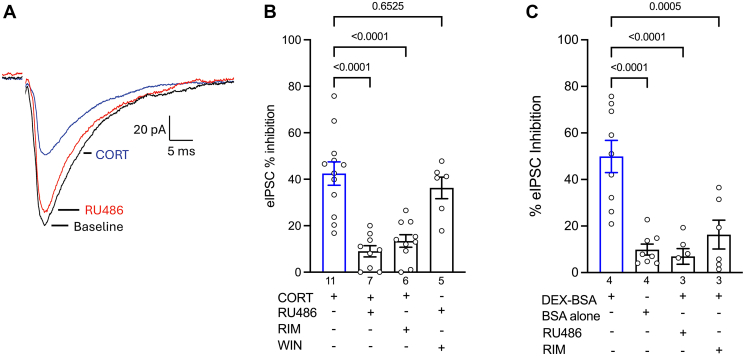
CORT-activated mbGRs indirectly inhibit GABA release via CB1R activation in vlPAG. (A) Representative eIPSC traces at baseline: NBQX, 5 *μ*M (black); CORT, 1 *μ*M (blue), and RU486, 3 *μ*M (red). (B) Inhibition of eIPSCs by CORT (1 *μ*M) is blocked by preincubation of aCSF solution containing RU486 (3 *μ*M) and RIM (3 *μ*M). The CB1R agonist WIN 55212-2 inhibits eIPSCs in the presence of RU486. One-way ANOVA F_(3,33)_ = 17.44, *P* < .0001. Dunnett’s post hoc test (*P* values on graph). (C) Percentage inhibition of eIPSCs by DEX:BSA (1 *μ*M) compared with BSA (1 *μ*M) alone, and DEX-BSA in the presence of RU486 (3 *μ*M) and RIM (3 *μ*M). One-way ANOVA F_(3,25)_ = 15.48, *P* < .0001. Dunnett’s post hoc test (*P* values on graph). Error bars represent the SEM, dots indicate individual recordings, and numbers represent the number of rats per experiment.

Reducing GABAergic neurotransmission within the vlPAG to disinhibit PAG projection neurons to the rostral ventromedial medulla is antinociceptive.^16^^,^^33^^,^^44^ We examined the ability of CORT to modulate GABAergic synapses in the vlPAG. CORT superfusion reduced eIPSC amplitudes within 5 minutes and the effect of CORT was reversed by the GR antagonist RU486 (3 *μ*M; Fig. 1, A and B). CORT-mediated inhibition of eIPSCs was similar for recordings from female and male rats (unpaired *t*-test, t_(19)_ = 0.42, *P* = .68, female: 12 cells from 8 rats; male: 9 cells from 9 rats), so data from both males and females were combined for all experiments. The rapid time course is similar to nongenomic rapid GR agonists signaling that is reversed by RU486 observed by other laboratories.^5^^,^^45^, ^46^, ^47^

To test whether CORT activation of mbGRs results in an increase in endocannabinoids, we also tested the ability of the CB1R antagonist RIM (3 *μ*M) to reverse the suppression of eIPSCs by CORT. Preincubation with the CB1R antagonist RIM (3 *μ*M) reduced the effect of CORT to the same extent as RU486 (Fig. 1B). In contrast, RU486 (3 *μ*M) did not block CB1R-mediated inhibition of eIPSCs observed in response to the cannabinoid agonist WIN 55212-2 suggesting that GR activation occurs upstream of CB1R effects. These results indicated that CORT-induced inhibition of eIPSCs occurs via activation of CB1Rs. Notably, the effect of CORT on glutamatergic eEPSCs was also assessed. However, CORT had minimal impact on eEPSC amplitudes (CORT 1 *μ*M: 5.4% ± 1.9% inhibition; 9 cells/6 rats).

In all experiments, the effect of CORT was observed quickly, 5–10 minutes after the drug superfusion, suggesting the involvement of mbGRs. To corroborate this, we tested a modified GR agonist DEX-BSA that cannot cross the membrane and specifically targets mbGRs. DEX-BSA (1 *μ*M) suppressed GABA release to a similar extent as CORT (1 *μ*M), confirming that CORT acts via mbGRs (Fig. 1C). BSA alone control experiments verified that BSA has minimal effects on eIPSCs. Furthermore, the effects of DEX-BSA were blocked in the presence of RU486 (3 *μ*M) and RIM (3 *μ*M; Fig. 1C), similar to results with CORT (Fig. 1B).

### 3.2 CORT prolongs CB1R activation by stimulating 2-AG synthesis

**Fig. 2:**
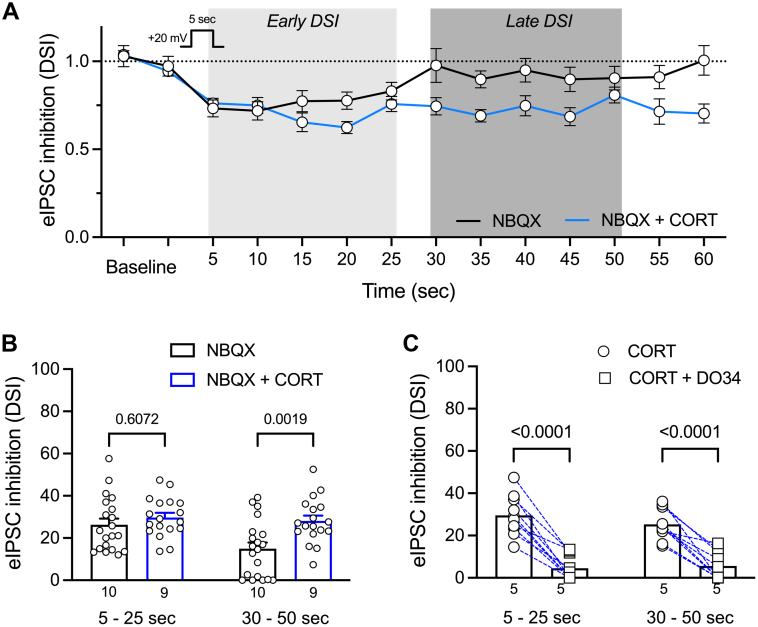
CORT prolongs CB1R activation by stimulating 2-AG synthesis. (A) Summary of DSI (+20 mV for 5 seconds) in vlPAG slices from naive rats without drug exposure (black line; *n* = 20 recordings from 10 rats) and after CORT 1 *μ*M (blue line; *n* = 18 recordings from 9 rats). (B) Quantification of inhibition of eIPSC amplitudes during early and late time windows (respectively after 5–25 and 30–50 seconds after the depolarization step). Two-way repeated-measures ANOVA; main effect of drug: F_(1,36)_ = 5.94, *P* = .02; main effect of time: F_(1,36)_ = 13.82, *P* = .0007; interaction: F_(1,36)_ = 7.77, *P* = .0084; Sidak’s multiple comparisons, *P* values on graph. (C) Quantification of inhibition of eIPSC amplitudes during early and late time windows on slices exposed to CORT 1 *μ*M with or without coexposure of DO34 1 *μ*M (*n* = 10 recordings from 5 rats). Two-way repeated-measures ANOVA; main effect of drug: F_(1,18)_ = 50.71, *P* < .0001; main effect of time: F_(1,18)_ = 1.81, *P* = .20; interaction F_(1,18)_ = 5.25, *P* = .034; Sidak’s multiple comparisons, *P* values on graph. Error bars represent SEM, dots indicate individual recordings, and numbers represent the number of rats represented per bar.

To determine the underlying mechanisms of CORT-induced inhibition of GABA release via CB1R activation, we directly measured the effects of CORT on endocannabinoid-mediated inhibition of GABA release using a DSI protocol. As previously observed in recordings from vlPAG neurons,^35^ the DSI protocol (+20 mV for 5 seconds) leads to a swift and temporary reduction in presynaptic GABA release mediated by CB1Rs, causing a brief suppression of eIPSC amplitudes before they return to baseline within 30 seconds. This result is consistent with other studies assessing DSI.^35^^,^^48^ Interestingly, inhibition of eIPSCs is prolonged in the presence of CORT superfusion (Fig. 2A). We assessed the maximal eIPSC inhibition over the first 5 eIPSCs (5–25 seconds after depolarization) and compared to the subsequent 5 eIPSCs (30–50 seconds), as in our prior study.^35^ During the early time window, the extent of DSI-initiated eIPSC inhibition was similar in the absence and presence of CORT. In contrast, during the late time window, although eIPSC amplitudes in the control group returned to baseline, CORT superfusion maintained prolonged inhibition (Fig. 2B).

Finally, to determine which endocannabinoid supports the prolonged effect of CORT, we tested DO34, an inhibitor of diacylglycerol lipases (DAGL) that synthesize 2-AG. DO34 is a dual DAGL*α*/*β* inhibitor that blocks 2-AG synthesis in the vlPAG.^35^ Additionally, DO34 was previously used to inhibit both DSI and depolarization-induced supression of excitation,^49^ supporting its relevance for our experimental paradigm. Superfusion of slices with DO34 (1 *μ*M) reduced CORT-mediated inhibition of eIPSCs during both the early and late phases of the DSI protocol (Fig. 2C). Taken together, these data indicate that CORT decreases GABA release in vlPAG via mbGR-induced synthesis of 2-AG.

### 3.3 CORT requires PKA activation to decrease GABA release

**Fig. 3:**
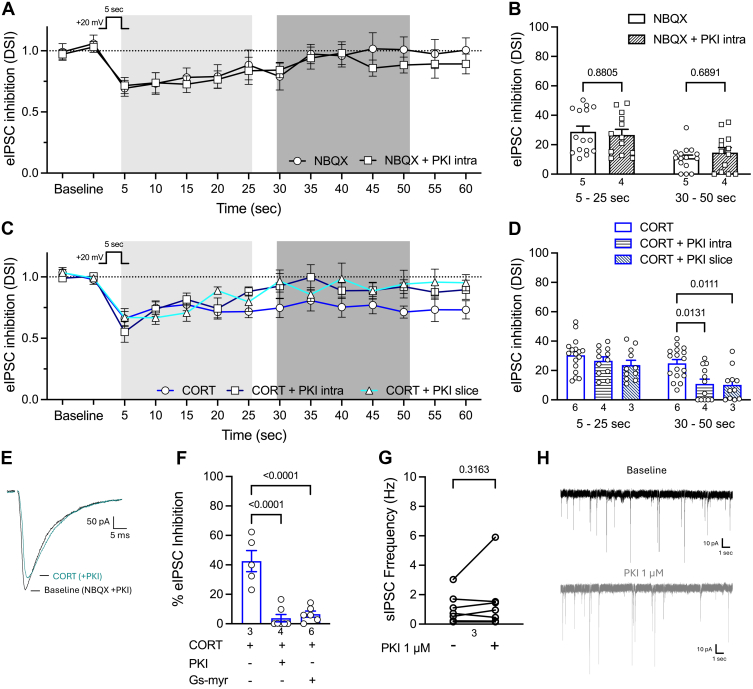
CORT stimulates DAGL activity via a PKA-dependent mechanism. (A) Summary of DSI (+20 mV for 5 seconds) from vlPAG recordings in the absence (white circles; *n* = 15 recordings from 5 rats) and presence of PKI 300 nM in the recording electrode (white squares; *n* = 13 recordings from 4 rats). (B) Quantification of inhibition of eIPSC amplitudes during early and late time windows. (C, D) Summary of DSI after 5–10 minutes bath application of CORT 1 *μ*M (circles; *n* = 17 recordings from 6 rats), with PKI 300 nM in the recording electrode (squares; *n* = 12 recordings from 4 rats), and with PKI 1 *μ*M added in perfusion solution with CORT (triangle; *n* = 11 recordings from 3 rats). Panels A–D were analyzed with two-way repeated-measures ANOVA; main effect of treatment: F_(4,63)_ = 3.14, *P* = .02; main effect of time: F_(1,63)_ = 53.24, *P* < .0001; interaction: F_(4,63)_ = 1.69, *P* = .16; Tukey post hoc test, *P* values on graphs. (E). Representative eIPSCs showing blockade of CORT-mediated inhibition after incubation of slice in PKI (1 *μ*M). (F) Compiled percent inhibition of eIPSCs by CORT in the absence and presence of PKI and a Gs peptide inhibitor (one-way ANOVA, F_(2,15)_ = 26.64, *P* < .0001; Dunnett’s post hoc test, *P* values on graph). (G) sIPSC frequencies are not different at baseline and after bath application of PKI 1 *μ*M from slices of naive rats (paired *t*-test, t_6_ = 1.09, *P* = .32). (H) Representative traces of sIPSCs in absence and presence of PKI. Error bars represent the SEM, dots indicate individual recordings, and numbers represent the number of rats represented per bar.

Malcher-Lopes et al^10^ demonstrated that mbGR activation in mouse neuroblastoma cells is linked to the cAMP-PKA pathway, suggesting the involvement of G*α*_s_ proteins and PKA. DAGL requires phosphorylation to convert diacylglycerol into 2-AG.^50^, ^51^, ^52^ Based on these findings, we coperfused CORT with PKI, an inhibitor of PKA and a G*α*_s_ inhibitor peptide (G_s_-myr). This peptide inhibitor, a myristoylated analog of the G*α*_s_ C-terminal domain, blocks interaction between G-protein-coupled receptors (GPCRs) and G*α*s proteins.^40^ First, the PKI was incorporated into our intracellular recording solution (PKI 300 nM) or directly coperfused with CORT + PKI 1 *μ*M. PKI (intrapipette or superfusion) did not affect DSI experiments in the absence of CORT (Fig. 3, A and B) indicating that DSI-mediated transient, early CB1R activation occurs independently of PKA activation. However, the prolonged eIPSC inhibition observed in DSI recordings from vlPAG neurons exposed to CORT (Fig. 3, C and D) was abolished when PKI was superfused over the slices or included in the pipette. Second, the effects of CORT on eIPSCs were blocked following incubation of slices with PKI (1 *μ*M) or Gs-myr (5 *μ*M; Fig. 3, E and F). Control experiments confirmed that PKI alone did not affect sIPSCs under basal conditions (Fig. 3, G and H) confirming that inhibition of PKA was not affecting GABA release directly.^53^ These findings indicate that prolonged CB1R activation by 2-AG depends on both G*α*_s_ and PKA activation.

## 4 Discussion

Here, we describe a mechanism by which CORT reduces GABAergic neurotransmission in the vlPAG by activating putative mbGRs within minutes of CORT administration. This effect is mediated through the synthesis of 2-AG and subsequent activation of CB1Rs, as evidenced by the reversal of CORT’s effects with the CB1R antagonist RIM and the DAGL inhibitor DO34 that blocks synthesis of 2-AG. CORT prolongs CB1R activation by 2-AG in a PKA-dependent manner, whereas early DSI-initiated CB1R activation remains independent of this pathway. These findings highlight the critical role of CORT-mbGR signaling in engaging endocannabinoid pathways to regulate GABA release in the vlPAG, providing insights into how stress hormones impact descending pain modulatory circuitry.

CORT is an important hormone regulating the HPA axis response to stress, as well as synaptic activity in various brain regions.^54^^,^^55^ Our previous study described an increase in 2-AG signaling and CB1R inhibition of GABA release in the PAG of inflamed animals treated with Complete Freud’s Adjuvant.^35^ Given that Complete Freud’s Adjuvant treatment increases CORT levels,^56^, ^57^, ^58^ we were interested in whether CORT plays a role in increasing 2-AG synthesis in the vlPAG. Our data reveal that bath application of CORT induces rapid inhibition of eIPSCs (within minutes). CORT inhibition of eIPSCs was reversed and prevented by a GR antagonist, confirming the presence of GRs in the vlPAG. Activation of GRs promote endocannabinoid synthesis in many brain areas.^5^^,^^7^^,^^45^^,^^59^, ^60^, ^61^, ^62^, ^63^ In order to directly measure the effect of CORT on endocannabinoids, we used a DSI protocol. DSI involves postsynaptic depolarization, leading to 2-AG synthesis via activation of DAGL.^64^, ^65^, ^66^, ^67^ We observed that the DAGL inhibitor, DO34, blocked CORT-mediated inhibition of GABA release, supporting the interpretation that CORT increases 2-AG synthesis. An increase in 2-AG levels could also be produced by inhibition of enzymes that degrade 2-AG, such as monoacylglycerol lipase (MAGL) and *α*/*β*-hydrolase domain-containing 6^24^ and this could contribute to the observed effect. However, inhibition of MAGL results in desensitization of CB1Rs in the vlPAG^35^ and similar results would be expected for inhibition of *α*/*β*-hydrolase domain-containing 6. Thus, inhibition of hydrolysis would not produce the observed CB1R-dependent prolongation of DSI in the presence of CORT.

CORT-mediated inhibition of presynaptic GABA release is blocked with CB1R antagonists suggesting that CORT-mediated inhibition of eIPSCs in the vlPAG is indirect via endocannabinoids acting on CB1Rs. Endocannabinoids are synthesized on demand and bind presynaptic CB1Rs inhibiting neurotransmitter release.^68^^,^^69^ Importantly, the GR antagonist did not affect direct activation of CB1Rs by the cannabinoid agonist WIN55212-2. This indicates that CORT modulates endocannabinoid signaling upstream of CB1R activation. This mechanism enables CORT to fine-tune neuronal activity in response to stressors via the endocannabinoid system.^6^^,^^70^ Although the rapid effects of CORT on GABAergic transmission in the vlPAG have not been previously examined, our results align with findings from other brain regions.^9^ For example, studies in the entorhinal cortex and basolateral amygdala demonstrate that CORT decreases the frequency of sIPSCs and miniature IPSCs without affecting amplitudes, suggesting that CORT decreases GABA release from presynaptic terminals.^13^^,^^71^ Additionally, field potential recordings reveal rapid CORT-induced suppression of local inhibitory networks in the somatosensory cortex.^72^

The rapid action of CORT on eIPSCs suggests involvement of mbGRs rather than genomic GRs which have longer time course (hours to days) between activation and signaling outcomes. Rapid nongenomic actions of cortocosterone and dexamethasone have been observed in several studies.^5^^,^^45^, ^46^, ^47^ However, there is a controversy whether mbGRs exist as the GR antagonist RU486 does not have binding affinity for mbGRs, at least in amphibian neuronal tissue^73^ and does not block fast glucocorticoid agonist effects in some studies,^62^ but does block in others.^5^^,^^9^^,^^45^, ^46^, ^47^^,^^74^ To confirm mbGR involvement, we tested a membrane-impermeable DEX-BSA conjugate which restricts dexamethasone activity to the cell surface. These experiments showed similar fast eIPSC inhibition compared with CORT, further confirming that CORT and DEX-BSA act on mbGRs rather than nuclear GRs. DEX-BSA-mediated inhibition was also blocked in the presence of GR and CB1R antagonists suggesting that CORT and DEX-BSA activate the same GRs.

Interestingly, we did not find evidence for an effect of CORT on glutamate release, even though there is evidence that glutamatergic afferents into the PAG express CB1Rs.^22^^,^^31^^,^^75^ Effects of endocannabinoids on glutamate synapses are observed readily in the presence of MAGL inhibitors or other neuromodulators that enhance endocannabinoid synthesis.^22^^,^^36^ This may be a function of CB1R expression on projections from specific brain regions.^32^ It is not known if CORT affects presynaptic glutamate release from these areas. Taken together, it is likely that mbGR-induced increases in 2-AG are spatially restricted to GABAergic afferents expressing CB1Rs in the vlPAG. Further studies examining the precise localization of mbGRs in the vlPAG are necessary to confirm this.

We hypothesized that CORT enhances DAGL activity via a PKA-dependent mechanism based on previous studies demonstrating that CORT increases PKA activity,^10^ and DAGL activity is modulated by PKA activity.^50^, ^51^, ^52^^,^^76^ Shonesy et al observed that PKA phosphorylation stimulates DAGL activity and this effect is prevented by the PKI.^51^ In our study, PKA inhibition with PKI blocked CORT-mediated prolonged DSI without affecting early DSI responses, suggesting a dual mechanism regulating endocannabinoid signaling: an early, PKA-independent phase and a sustained, PKA-dependent phase. Notably, just delivering the PKI peptide directly to the postsynaptic neuron through the recording pipette was effective at inhibiting the late phase CORT-mediated prolongation of DSI. Bath application of the inhibitor peptide did not directly affect sIPSC frequency or amplitude, indicating that inhibition of PKA did not directly affect presynaptic GABA release. Although we can only speculate about CORT-mediated phosphorylation of DAGL due to a lack of direct measurements of phosphorylation, our results suggest that PKA activation is specifically required for the ability of CORT to increase 2-AG levels. Additionally, although the duration of DSI can also be modulated by metabotropic glutamate receptors,^77^ the lack of effect of CORT on glutamate release in our experiments suggests a minimal role of metabotropic glutamate receptors in the effects of CORT in the vlPAG.

We also investigated the G-protein signaling pathway used by mbGRs to activate PKA. Growing evidence suggests that at least some of the rapid glucocorticoid actions are mediated by GPCRs and downstream intracellular signaling cascades. An early study examining binding of radiolabeled glucocorticoids to membrane fractions of new brainstem tissue demonstrated suppression by GTP-*γ*S, indicating that glucocorticoids bind to one or more GPCRs.^78^ Considering the putative role of CORT in increasing PKA activity, we coperfused CORT with a peptide inhibitor capable of blocking G*α*_s_ protein binding to GPCRs.^40^^,^^79^^,^^80^ The G*α*_s_ peptide inhibitor prevented CORT inhibition of eIPSCs and suggested that the putative mbGRs are coupled to G*α*_s_ proteins in the vlPAG. These results align with previous findings that rapid glucocorticoid-induced synthesis and release of endocannabinoids is mediated by G*α*_s_ activation in the paraventricular nucleus.^10^^,^^62^

In summary, CORT-mediated activation of 2-AG synthesis prolongs DSI-mediated endocannabinoid inhibition of presynaptic GABA release in the vlPAG. Inhibiting GABAergic neurotransmission in the vlPAG leads to activation of the descending pain modulatory pathway.^16^^,^^33^ Rapid, on-demand mobilization of CORT could support the stress-induced analgesia produced by opioids.^25^^,^^81^, ^82^, ^83^ Extended CORT elevation may be maladaptive by prolonging endocannabinoid signaling, shifting from an on-demand mechanism to a sustained modulation of synaptic activity that would dampen the endocannabinoid system’s adaptive capacity, potentially contributing to dysregulation in stress-related disorders such as depression and chronic pain. Future studies should further investigate the CORT-endocannabinoid interactions in various chronic pain models to understand the role of sustained endocannabinoid modulation in the vlPAG.

## Conflict of interest

The authors declare no conflicts of interest.
